# Supplementary material for: Prognostic Value of Resistance Proteins in Plasma Cells from Multiple Myeloma Patients Treated with Bortezomib-Based Regimens
Source: J Clin Med. 2021 Oct 28;10(21):5028. doi: 10.3390/jcm10215028 (PMC8584776; doi:10.3390/jcm10215028)
Supplement: Supplementary file 1 [file jcm-10-05028-s001.zip › jcm-1359304-supplementary.pdf]

Supplementary Captions.

Table S1. Normalized protein expression values for all samples and most important clinical variables.

Table S2. Normalized protein levels according to ISS stage. Data are presented as mean and SD.

Table S3. Normalized protein levels according to previous treatment. Data are presented as mean and SD.

Table S4. Normalized protein levels according to anemia at the diagnosis. Data are presented as mean and SD.

Table S5. Protein levels according to hypercalcemia at the diagnosis. Data are presented as mean and SD.

Table S6. Normalized protein levels according to renal failure at the diagnosis. Data are presented as mean and SD.

Table S7. Normalized protein levels according to bone disease at the diagnosis. Data are presented as mean and SD.

Table S8. Normalized protein levels according to the response to bortezomib-based chemotherapy according to the IMWG criteria. Data are presented as mean and SD.

Table S9. Normalized protein levels according to refractoriness to bortezomib. Data are presented as mean and SD.

Table S10. The optimal cutpoints for protein levels determined by Cutoff Finder.

Table S11. Univariate Cox regression analyses of dichotomized protein levels for progression-free survival for patients without previous treatment (N=29).

Table S12. The optimal cutpoints for protein levels determined by Cutoff Finder for PFS of patients without previous treatment (N=29).

Table S1. Normalized protein expression values for all samples and most important clinical variables.

Patient ID	POMP normalized	PSMB5 normalized	NRF2 normalized	XBP1 normalized	cMAF normalized	MAFB normalized	Refractoriness	ISS	Previous treatment	Progression	PFS	OS	Death	ASCT
20	0.151273885	1.51050955	0.014996815	0.430254777	0.190796178	1398.08917	0		0	0	19.4666667	19.4666667	1	1
21	0.170496894	1.62360248	0.020068323	0.488819876	0.214192547	1751.5528	0	III	0	1	12.4666667	39.5	0	1
25	0.21986755	2.32582781	0.014536424	0.42218543	0.204470199	2009.93377	0	I	0	0	35.0333333	35.0333333	0	1
26	0.155095541	0.161783439	0.018904459	0.458917197	0.185127389	1668.78981	0	II	0	0	35.3666667	35.3666667	0	1
27	0.14760479	1.46586826	0.014350299	0.432934132	0.190658683	2170.65868	1	I	1	1	4.7	31.2666667	1	0
28	0.103492063	1.41650794	0.01404127	0.427301587	0.181968254	1311.11111	0	I	0	1	32.4	35.9666667	0	1
30	0.200928793	1.7498452	0.018690403	0.460990712	0.20377709	1888.54489	0	III	0	1	7.66666667	8.06666667	1	0
33	0.190909091	1.43039773	0.01615625	0.472443182	0.161477273	1806.81818	0	II	0	1	36.3	36.3	0	1
34	0.151446945	1.35948553	0.017263666	0.433440514	0.17829582	1279.74277	0	II	1	1	10.8666667	18.6333333	1	1
36	0.172755418	2.02352941	0.018306502	0.492879257	0.237832817	1622.29102	0	III	0	1	1.2	34.9666667	0	1
37	0.143086817	1.71028939	0.015717042	0.464951768	0.221028939	1559.48553	0	III	0	1	4.6	35.6666667	0	1

38,0.155974843,1.85597484,0.015993711,0.435849057,0.224433962,1783.01887,0,II,0,1,14.2666667,44.3333333,0,1
40,0.161587302,1.75809524,0.018876191,0.464126984,0.222031746,1850.79365,0,I,0,1,33.2,34.1333333,0,1
41,0.142507645,1.53639144,0.017810398,0.455963303,0.217492355,1868.50153,0,III,0,1,11.7666667,13.7666667,1,0
43,0.134202899,1.13942029,0.011295652,0.42,0.211768116,1321.73913,1,III,0,1,3.76666667,21.9333333,1,0
52,0.205740181,2.00845921,0.015755287,0.479154079,0.177915408,1477.34139,1,III,0,1,1.03333333,12.4,1,0
56,0.204746835,1.35727848,0.016237342,0.468987342,0.181297468,1462.02532,1,III,0,1,1.63333333,12.3666667,1,0
58,0.153416149,1.71149068,0.015726708,0.464596273,0.19826087,1975.15528,0,II,1,1,12.5666667,42.0666667,0,0
61,0.14925816,1.28724036,0.016130564,0.446884273,0.199910979,1727.00297,1,III,0,1,3.76666667,21.9333333,1,0
64,0.166984127,1.73396825,0.017215873,0.459047619,0.213714286,1946.03175,1,II,1,1,7.06666667,17.7333333,1,0
65,0.16,1.65016393,0.019334426,0.46852459,0.234360656,1931.14754,0,III,0,1,13.7,26.9666667,1,0
73,0.139263804,1.45214724,0.017901841,0.432822086,0.189079755,1524.53988,0,I,0,1,29.5,56.7666667,0,1
74,0.164240506,1.51297468,0.018012658,0.461392405,0.181044304,2212.02532,0,III,0,1,34.8,59.3333333,1,0
78,0.175609756,1.50060976,0.016689024,0.418902439,0.211158537,1896.34146,1,I,1,1,1.03333333,1.26666667,1,0
83,0.160909091,1.92818182,0.014227273,0.472424242,0.221090909,1466.66667,0,III,0,1,9.83333333,11.5333333,1,0
84,0.142207792,1.71525974,0.018525974,0.474675325,0.244383117,1142.85714,0,III,1,1,13.2666667,16.0666667,1,1
85,0.146290801,1.32967359,0.017186944,0.39851632,0.188071217,1703.26409,1,III,1,1,8.3,31.0333333,0,1
86,0.205571031,1.72618384,0.01732312,0.369637883,0.228022284,1637.88301,0,III,0,1,20.3,27.0333333,1,1
89,0.1678125,1.39125,0.0172375,0.4509375,0.1820625,2243.75,0,I,0,1,29.1666667,49.4666667,1,1
90,0.173114754,1.9652459,0.020839344,0.423934426,0.225737705,2540.98361,0,II,0,1,8.16666667,21.0666667,1,0
91,0.137650602,1.87138554,0.016409639,0.445481928,0.201174699,1939.75904,0,III,1,1,5.13333333,28.2333333,0,0
92,0.135060976,1.47042683,0.016121951,0.430792683,0.177835366,1637.19512,0,I,0,1,10.5,29.7666667,1,0
93,0.135504886,1.62801303,0.016276873,0.486644951,0.181335505,1657.98046,1,,0,1,12.0333333,33.9666667,0,0
94,0.205475504,2.00864553,0.017507205,0.440345821,0.214005764,2285.30259,0,III,0,1,11.7666667,12.0333333,1,0
101,0.214144737,2.13651316,0.016411184,0.422039474,0.226348684,1894.73684,1,I,0,1,22.6666667,23.2666667,0,0
114,0.149262537,1.33362832,0.012386431,0.413274336,0.154100295,1336.28319,1,I,0,1,1.73333333,1.83333333,0,0
115,0.113489736,1.39618768,0.017548387,0.378005865,0.218856305,973.607038,1,I,1,1,3.03333333,39.1333333,1,1
118,0.129216867,1.61295181,0.013593374,0.402409639,0.172379518,1228.91566,0,,1,1,11.6333333,20.7,0,1
119,0.206461538,1.972,0.019187692,0.426153846,0.203292308,2089.23077,1,I,0,1,10.5,29.7666667,1,0

Table S2. Normalized protein levels according to ISS stage. Data are presented as mean and SD.

Variable	ISS I/II	SD	ISS III	SD.	*p*-value
**POMP**	0.16	0.03	0.17	0.03	0.6322
**PSMB5**	1.54	0.45	1.68	0.28	0.3641
**NRF2**	0.017	0.002	0.017	0.002	0.9069
**XBP1**	0.44	0.02	0.45	0.03	0.1094
**cMAF**	0.20	0.02	0.21	0.02	0.0383
**MAFB**	1757.25	390.04	1716.23	293.05	0.7262

Table S3. Normalized protein levels according to previous treatment. Data are presented as mean and SD.

Variable	No previous treatment	SD	Previous treatment	SD	*p*-value
**POMP**	0.17	0.03	0.15	0.02	0.0350
**PSMB5**	1.62	0.40	1.57	0.19	0.4121
**NRF2**	0.019	0.002	0.016	0.002	0.6760
**XBP1**	0.45	0.03	0.43	0.03	0.1113
**cMAF**	0.20	0.02	0.20	0.02	0.9843
**MAFB**	1757.41	310.81	1625.63	426.24	0.3009

Table S4. Normalized protein levels according to anemia at the diagnosis. Data are presented as mean and SD.

Variable	HB ≥10 g/dL at diagnosis	SD	HB <10 g/dL at diagnosis	SD	*p*-value
**POMP**	0.17	0.03	0.16	0.03	0.2114
**PSMB5**	1.61	0.42	1.61	0.25	0.8678
**NRF2**	0.017	0.002	0.017	0.002	0.5875
**XBP1**	0.44	0.03	0.44	0.03	0.9918
**cMAF**	0.199	0.02	0.21	0.02	0.2262
**MAFB**	1737.64	345.26	1734.92	336.98	0.9813

Table S5. Protein levels according to hypercalcemia at the diagnosis. Data are presented as mean and SD.

Variable	Calcium ≤2.5 mmol/L at diagnosis	SD	Calcium >2.5 mmol/L at diagnosis	SD	*p*-value
**POMP**	0.16	0.03	0.17	0.03	0.6292
**PSMB5**	1.60	0.41	1.65	0.21	0.6292
**NRF2**	0.016	0.002	0.018	0.002	0.5875
**XBP1**	0.44	0.03	0.45	0.02	0.9918
**cMAF**	0.20	0.02	0.20	0.02	0.2262
**MAFB**	1663.82	333.04	1915.38	289.76	0.9813

Table S6. Normalized protein levels according to renal failure at the diagnosis. Data are presented as mean and SD.

Variable	Creatinine ≤2 mg/dL at diagnosis	SD	Creatinine >2 mg/dL at diagnosis	SD	*p*-value
**POMP**	0.16	0.03	0.18	0.03	0.2784
**PSMB5**	1.60	0.38	1.68	0.11	0.4904
**NRF2**	0.017	0.002	0.018	0.0006	0.1488
**XBP1**	0.45	0.03	0.44	0.05	0.7213
**cMAF**	0.20	0.02	0.21	0.03	0.2588
**MAFB**	1738.56	330.58	1720.33	451.05	0.9203

Table S7. Normalized protein levels according to bone disease at the diagnosis. Data are presented as mean and SD.

Variable	Without bone disease	SD	Bone disease	SD	*p*-value
**POMP**	0.16	0.03	0.16	0.03	0.5273
**PSMB5**	1.62	0.25	1.60	0.44	0.8772
**NRF2**	0.017	0.002	0.017	0.002	0.8625
**XBP1**	0.45	0.02	0.44	0.04	0.7093
**cMAF**	0.20	0.02	0.20	0.02	0.4858
**MAFB**	1772.42	330.95	1677.26	356.10	0.3936

Table S8. Normalized protein levels according to the response to bortezomib-based chemotherapy according to the IMWG criteria. Data are presented as mean and SD.

Variable	≥VGPR	SD	<VGPR	SD	*p*-value
**POMP**	0.16	0.03	0.17	0.03	0.3994
**PSMB5**	1.58	0.41	1.64	0.31	0.8107
**NRF2**	0.018	0.002	0.016	0.002	0.0167
**XBP1**	0.45	0.02	0.44	0.03	0.1234
**cMAF**	0.20	0.02	0.20	0.02	0.6520
**MAFB**	1761.34	374.04	1691.29	319.62	0.5320

Table S9. Normalized protein levels according to refractoriness to bortezomib. Data are presented as mean and SD.

Variable	Sensitive	SD	Refractory	SD	*p*-value
**POMP**	0.16	0.03	0.17	0.03	0.6819
**PSMB5**	1.63	0.38	1.56	0.31	0.1570
**NRF2**	0.017	0.002	0.016	0.002	0.0863
**XBP1**	0.45	0.03	0.44	0.03	0.1840
**cMAF**	0.20	0.02	0.20	0.02	0.3194
**MAFB**	1752.49	346.26	1665.87	342.37	0.4644

Table S10. The optimal cutpoints for protein levels determined by Cutoff Finder.

	**PFS**			**OS**		
**Protein**	Cut-off point	N below cut-off point	N above cut-off point	Cut-off point	N below cut-off point	N above cut-off point
**POMP**	0.1503	15	24	0.1729	28	11
**PSMB5**	1.525	17	22	1.713	24	15
**NRF2**	0.01786	28	11	0.01786	28	11
**XBP1**	0.4267	11	28	0.4612	26	13
**cMAF**	0.2078	22	17	0.1899	14	25
**MAFB**	1638	16	23	1501	11	28

Table S11. Univariate Cox regression analyses of dichotomized protein levels for progression-free survival for patients without previous treatment (N=29).

**Variables**	**PFS**				
	Coefficient	*p*-value	HR	95% CI	
				lower	higher
**High POMP**	-0.233	0.2790	0.627	0.269	1.460
**High PSMB5**	0.308	0.1530	1.851	0.796	4.308
**High NRF2**	-0.157	0.4547	0.731	0.321	1.664
**High XBP1**	0.184	0.3799	1.45	0.635	3.287
**High cMAF**	0.352	0.0988	2.020	0.876	4.658
**High MAFB**	-0.403	0.0554	0.446	0.195	1.019

Table S12. The optimal cutpoints for protein levels determined by Cutoff Finder for PFS of patients without previous treatment (N=29).

PFS			
**Protein**	Cut-off point	N below	N above
**POMP**	0.1532	10	19
**PSMB5**	1.525	12	17
**NRF2**	0.01614	11	18
**XBP1**	0.4628	19	10
**cMAF**	0.2081	16	13
**MAFB**	1638	12	17
